# The Sun/Moon Illusion in a Medieval Irish Astronomical Tract

**DOI:** 10.3390/vision3030039

**Published:** 2019-08-07

**Authors:** Helen E. Ross

**Affiliations:** Psychology, University of Stirling, Stirling FK9 4LA, UK; h.e.ross@stir.ac.uk

**Keywords:** sun illusion, moon illusion, medieval science, atmospheric refraction, Messahala, flat Earth, spectacles

## Abstract

The Irish Astronomical Tract is a 14th–15th century Gaelic document, based mainly on a Latin translation of the eighth-century Jewish astronomer Messahala. It contains a passage about the sun illusion—the apparent enlargement of celestial bodies when near the horizon compared to higher in the sky. This passage occurs in a chapter concerned with proving that the Earth is a globe rather than flat. Here the author denies that the change in size is caused by a change in the sun’s distance, and instead ascribes it (incorrectly) to magnification by atmospheric vapours, likening it to the bending of light when looking from air to water or through glass spectacles. This section does not occur in the Latin version of Messahala. The Irish author may have based the vapour account on Aristotle, Ptolemy or Cleomedes, or on later authors that relied on them. He seems to have been unaware of alternative perceptual explanations. The refraction explanation persists today in folk science.

## 1. Early Explanations of the Sun or Moon Illusion

The sun/moon illusion is the apparent enlargement of the celestial bodies when near the horizon compared with their appearance when higher in the sky. Early authors usually referred to it as the sun illusion, and modern authors as the moon illusion. The effect was often ascribed to some type of refraction by the atmosphere—but this cannot be correct, because magnification only occurs when looking from a thin to a denser medium (such as air to water), but not the other way around. Refraction does have the effect of making the image of the sun oval, and slightly smaller, when near the horizon. The various accounts were reviewed by Plug and Ross [1] and Ross and Plug [2] and the main points are summarised here. Aristotle (fourth century BC) gave an obscure account, comparing the magnification to reflection in an enlarging mirror, or to refraction of light passing between media of different optical density. Similar statements were made by Posidonius (ca. 135–50 BC) and Seneca (ca. 3 BC–AD 65). Ptolemy was clearer about refraction in his book on astronomy, the *Almagest* (ca. AD 142), as was Cleomedes (early third century AD): Both authors compared the horizon enlargement to the magnification of objects seen under water. However, in other passages both authors state that perceived size does not correspond to image size, but rather to linear size through the geometrical principle of size-distance invariance. In his later book the *Optics* (ca. AD 170), Ptolemy explained underwater enlargement as due to angular magnification combined with the same (apparent) distance as in air. Cleomedes elaborated on the importance of apparent distance and claimed that a misty atmosphere (aerial perspective) made the sun appear further away near the horizon, and thus larger. Ibn al-Haytham (ca. 1039) criticised Ptolemy’s accounts of atmospheric refraction and attempted to use the correct direction of refraction by stating that the eye is in the thinner medium and the object in the denser. Refraction accounts were repeated without criticism by Arab astronomers, and by the British scholars Alexander Neckam and John of Sacrobosco in the thirteenth century. Atmospheric refraction was not clearly understood until about the sixteenth century.

The importance of apparent distance was elaborated by Ibn al-Haytham in much more detail. He described the flattened-dome account in his *Optics* (ca. 1039), and it was repeated by the thirteenth-century writers Roger Bacon, John Pecham and Witelo. On this theory the dome of the sky appears flattened because intervening objects expand the apparent distance towards the horizon, and the apparent size of the celestial bodies expands with the apparent distance. John of Sacrobosco repeated a version of this based on the ninth-century Arab astronomer al-Farghani, but in this version the flattened dome had a physical reality, and the midday sun was actually closer than at sunrise or sunset. This should produce the opposite effect to the sun illusion.

The experimental study of the illusion did not begin until the early 1900s. Experiments have shown that several factors can contribute to the illusion, but the most important is the sight of the terrain which sets the scale for enlarging the apparent size of distant objects. There is currently no final agreed explanation [3]. The popular press today continues to report the refraction account [4], or sometimes the apparent distance account.

## 2. The Irish Astronomical Tract

The Irish Astronomical Tract exists as three manuscripts in Dublin [5]. These date from about the late fourteenth century and are all in different hands. The original was probably written in the mid fourteenth century. Since it refers to spectacles, it cannot be earlier than about 1325 [6] or 1320 [7]. It has been translated into English by Power [6] and Williams [5]. The Tract consists mainly of a translation into Irish of a Latin version of Messahala’s treatise on astronomy (*De scientia motus orbis*—*On the knowledge of the motion of the orb*). Messahala (ca. 740–815) was a Jewish scholar, versed in Arabic learning, whose Arabic text was translated into Latin by Gerard of Cremona, and reached the West in the twelfth century. His book was based mainly on Ptolemy’s astronomy and Aristotle’s physics, and it became very popular in the middle ages. Most of his work survives only in Latin, and the Irish manuscript is the only known translation into a vernacular language [8]. His writings have been translated into English by Dykes [9], where the treatise on astronomy has 28 sections. The Irish Tract has 40 (or 41) chapters, 27 (or 28) of which correspond approximately to sections of Messahala. Thirteen chapters in the Tract are not present in the Latin version of Messahala. According to Williams [5] (p. 26) they are probably derived from Aristotle’s *De Caelo* and *Meteorologica* and *De Plantis* (fourth century BC) and from other classical authors such as Eratosthenes (276–194 BC), Posidonius (135–51 BC), Strabo (63 BC–AD 23), Pliny the Elder (AD 23–79), Cleomedes (ca. AD 200) and the early-medieval Isidore of Seville (AD 560–636). Chapter 7, in which the sun illusion is discussed, is one of the chapters that is not a direct translation of Messahala. 

## 3. Contents of Chapter 7: ‘The Rotundity of the Earth and the Knowledge of Day and Night’

The author’s main purpose was to prove that the Earth is a globe and not flat. We give only a summary of his arguments but quote the passage about the sun illusion in full. 

The Earth is a globe and not flat. If it were flat, water would not run from place to place, but would form a large sea on its surface. Additionally, if it were flat, you should be able to see the same stars from the north and south parts; but the convexity of the Earth conceals the sky behind you and reveals different stars in front of you. As the sun travels round the Earth it illuminates half of it, causing daylight, while the other half is in darkness. The Earth is a globe, and wherever people stand, their heads are up and their feet down. If the Earth were flat, the sun would appear small when rising in the distance, and large when nearer overhead; but the sun appears of equal size in all directions. 

**Figure 1:**
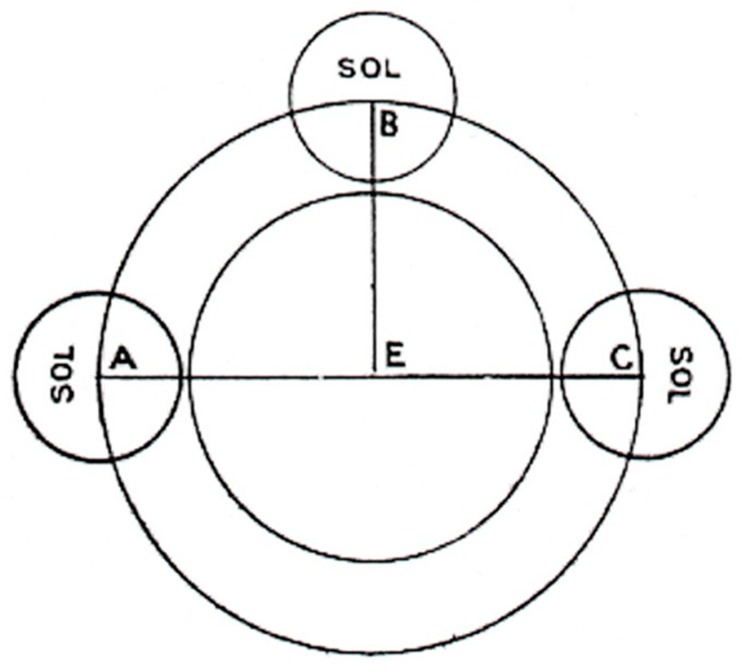
The Earth is a globe and the sun (SOL) is equidistant at all locations. (Reproduced from Williams [5]).

A diagram can be constructed (Figure 1) to show the Earth as a globe and the sun travelling around it and equidistant from it at all locations. E is the centre of the globe, A is the west, B the top and C the east. The sun (SOL) is represented by a small circle, which has the same size at all locations.

The author goes on to deal with the objection that the sun appears larger when rising or setting: “Whoever should declare as an argument against this that the sun appears distinctly larger when rising or setting than it does at the highest point at mid-day, and that it is understood from this that it is further away at mid-day, than when it is in those other quarters, and that this proves that the earth is a level plane without convexity, I reply to him appropriately, in giving a solution for that argument, that that often happens, but not always, and when it does happen the reason is—when the sun is rising or setting, it draws up the moisture and the rain and black wet vapours rise to a great height between us and it, and then, when we look at the sun, that mist which is seen broadens and amplifies the sphere of vision within it, therefore, according to the denseness and materiality of that mist, does the sun appear larger through it, than it would appear without that mist being present. As the day advances, and the sun is at its highest point of the firmament with no mist between us and it, then we see it with its own proper size.

The example is clearly illustrated in the case of the naked person under water, because he appears larger to the sight under water than out of water; although there is no proof in that, except the fact of the wet dense water spreading and amplifying the sight, and preventing it from passing directly and naturally towards the person. The same reason is the cause of an object appearing larger and thicker through glass than otherwise. Consequently old people, who are losing their sight so that they cannot read small letters, use glass spectacles to magnify the letters they read, and for the same reason the sun appears larger in the early morning and in the evening than at mid-day, as I have mentioned.” (Translation Williams [5]).

**Figure 2:**
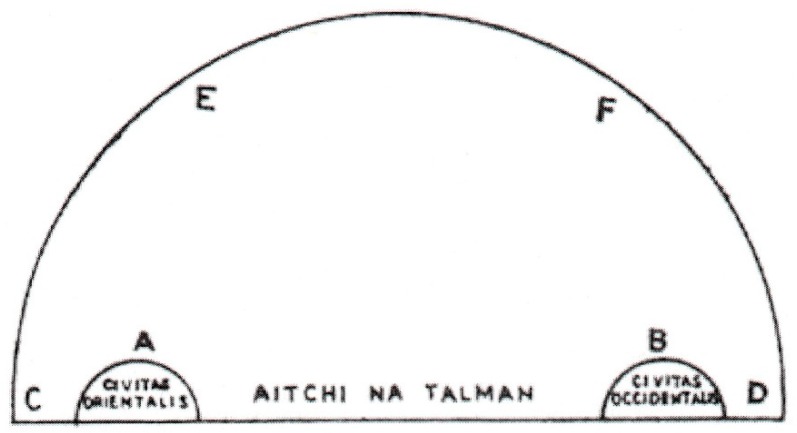
Flat Earth showing places in the east (**A**) and west (**B**). (Reproduced from Williams [5]).

The text goes on to add that if the Earth were a level plane, people living in the east should see the sun rising in the east as a large mass but setting in the west as a smaller mass; but people living in the west should see it small when rising but large when setting. Similarly, for people living in the east, the first half of the day should seem shorter than the second half; and the other way around for people living in the west. A diagram illustrates this point (Figure 2). A straight line shows places on the surface of the Earth (AITCHI NA TALMAN in Irish). A represents an eastern community (CIVITAS ORIENTALIS in Latin) and B a western community (CIVITAS OCCIDENTALIS in Latin); C is sunrise and D sunset; E is mid-day for the eastern place and F mid-day for the western place. For the eastern place, the time for the sun to reach to mid-day (C to E) is much shorter than the time for it to reach sunset (E to D); and the other way around for the western place. This is clearly untrue, and the sun sets at different times in different places.

## 4. Comments on the Text

The Irish author’s main purpose was to discredit the belief that the Earth was flat, and to show that it was a globe. Belief in a flat Earth was outdated, and most people knew that it was a globe [10]. Pliny in his *Natural History*, 2.64 [11] said that it was common knowledge, and Isidore made similar comments (*Etymologies* III. xxxii; XIV, 1) [12]. The Tract repeats some stock arguments against a flat Earth. For example, Martianus Capella (AD 360–428) wrote a popular textbook [13] which included a chapter on ‘geometry’ (measuring the Earth). He made the points (sections 590–595) that the rising and setting times of the stars, and the hours of daylight, vary on different parts of the Earth; that constellations can be hidden by the curvature of the Earth; and that eclipses of the sun or moon can be seen in some parts of the world and not in others. Martianus was probably copying Pliny (*Natural History*, 2, 162) [11]. The Irish author probably also used other parts of Messahala, who argued that water flows round the orb of the Earth (Section 5) and that the celestial bodies rise and set at different times for cities on different parts of the Earth (Section 11). The diagrams in the Irish Tract for this chapter were not directly copied from any of the Latin texts of Messahala and may have been added by the author to help students understand the text [8]. Figure 1 is well drawn and is similar to other diagrams in the Messahala text. Figure 2, on the other hand, is less clearly written and contains both Latin and Irish text. The diagram reproduced here has a corrupt text and should probably read ‘line aigthe na talman’ meaning ‘line of the surface of the Earth’ (Patricia Kelly, personal communication). The diagram is also unconventional by modern standards in placing east to the left and west to the right, which is the opposite of Figure 1; it does, however, agree with the order suggested by the letters in the text, and it also agrees with the order in some diagrams in Messahala [9] (pp. 256, 266).

Many later authors continued to assert that the Earth was a globe. These included the Venerable Bede (c. 672–735) [14] (pp.91–93), the Irish saint Fergal or Vergilius of Salzburg (700–784) [15], and John of Sacrobosco who wrote his tract around 1230 [16] (p. 119). 

The discussion of the sun illusion was of secondary interest to the author, who only brought it in to explain the apparent enlargement of the sun near the horizon. It was essential for the argument against a flat Earth that the sun should be at the same distance and thus subtend the same angular size at all locations in the sky. A brief explanation of the apparent enlargement at the horizon would be sufficient for the purpose, and the popular refraction account probably seemed adequate. There was no need to expand on alternative explanations.

The author of the Tract was not alone in ignoring other explanations of the sun illusion. Like many authors, he simply copied the most common account found in other textbooks without reading more widely. Even today most people believe that the enlargement is an optical effect caused by refraction, and this is repeated in popular science notes [4].
